# An Unusual Combination of Three Rare Complications: Pleuro-Pancreatic Fistula, Chylous Ascites, and Renal Vein Thrombosis, in a Case of Acute Severe Pancreatitis

**DOI:** 10.1055/s-0039-1700807

**Published:** 2019-11-21

**Authors:** Tanweerul Huda, Anjaly Mohan, Mohammad Masoom Parwez, Bharati Pandya

**Affiliations:** 1 Department of General Surgery, All India Institute of Medical Sciences, Bhopal, Madhya Pradesh, India

**Keywords:** acute pancreatitis, pleuro-pancreatic fistula, pancreatic ascites, chylous ascites, renal vein thrombosis

## Abstract

**Background** Acute pancreatitis is fraught with a variety of complications, which account for the mortality associated. Our case had a fulminant course, with three rare, near-fatal complications and was successfully managed conservatively. Pleural effusion due to pleuro-pancreatic fistula is uncommon, seen in only 1% cases, of which right-sided effusions are rarer still. Management modalities include conservative, endoscopic, and surgical options. Chylous ascites is an extremely rare complication of pancreatitis and is managed with high protein, low lipid diet, restricted to medium-chain triglycerides (MCTs). Extra-splanchnic venous thrombosis is uncommon in pancreatitis, and isolated renal vein thrombosis is very rare.

**Case Presentation** A 34-year-old, chronic alcoholic male, presented to the outpatient department (OPD) in a state of shock and respiratory distress. Chest radiograph showed massive right-sided pleural effusion. The pleural fluid was hemorrhagic with markedly elevated amylase levels, and contrast-enhanced computed tomography (CECT) confirmed the presence of a right-sided pleuro-pancreatic fistula. Left renal vein thrombosis was also noted. The patient improved with chest drain, intravenous (IV) octreotide, and anticoagulants. Subsequently, he developed hemorrhagic pancreatic ascites, which later turned chylous. This was managed with dietary modifications. The patient had a prolonged recovery but was finally discharged after 45 days.

**Conclusion** It is a challenge managing the various complications of acute severe pancreatitis. We describe this case to emphasize maintaining a high sensitivity for timely diagnosis and appropriate addressal of all the complications for better patient outcomes.

The course of acute pancreatitis is often complicated by a variety of systemic as well as local events that contribute to mortality and morbidity. These complications often pose a challenge in the resolution of the underlying disease process, and it is crucial to individualize the management as per their merits in each patient. Pleural effusion is associated with 4 to 17% of the cases of pancreatitis, 1 of which right-sided pleural effusion is rarer still, and can pose as a diagnostic dilemma. Chylous ascites can exceptionally also be noted in cases of pancreatitis when extensive cytolysis and necrosis affect the lymphatic channels. Venous thrombosis in the pancreas is most often encountered in the splanchnic veins and the splenic vein. Renal vein thrombosis has been diagnosed in rare cases mainly with associated inferior vena cava (IVC) thrombosis. Isolated renal vein thrombosis in pancreatitis is extremely rare. 2

Our patient had a complicated clinical course with all three of the near-fatal complications and was intensively managed successfully on conservative lines.

## Case Presentation

A 34-year-old male, presented to the OPD in a state of shock with no urine output for over 24 hours, acute onset of shortness of breath, and epigastric pain radiating to the back, of 3 days duration. He was hospitalized elsewhere and referred to us on deterioration. He had no comorbidities but had a history of chronic alcohol abuse for the past 4 years.

**Fig. 1:**
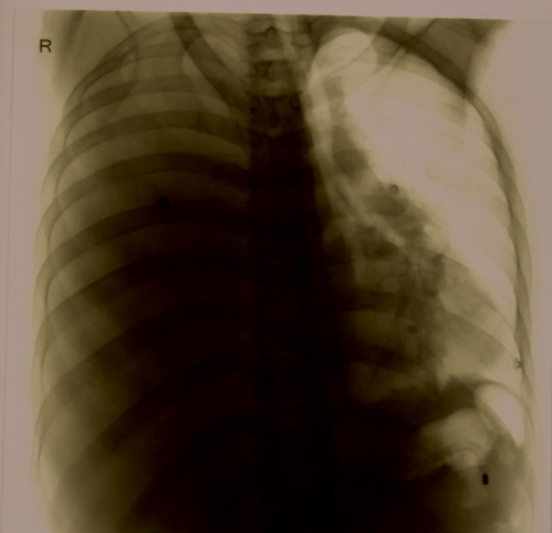
Chest radiograph.

**Fig. 2:**
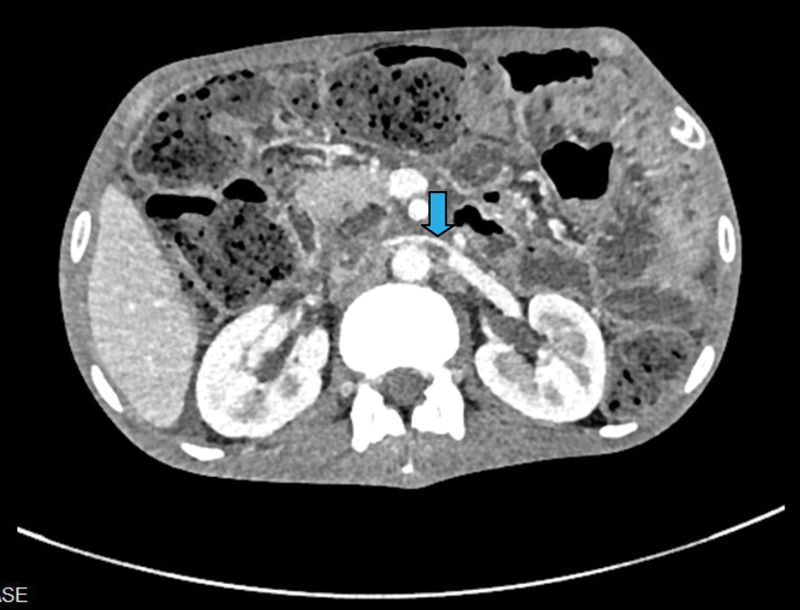
Left renal vein thrombus (blue arrow).

On examination, he was pale, emaciated, weighing only 29 kg and in hemodynamic shock. He had a dull percussion note, and diminished breath sounds all over the right chest. An abdominal examination showed mild epigastric tenderness, with no evidence of free fluid and absent bowel sounds. Investigations revealed hemoglobin of 10.6 g%, leukocyte counts were elevated to 19,450/mm ^3^ with neutrophil predominance. Liver functions and renal parameters were mildly deranged, and amylase was 1,253 U/L and lipase 3,810 U/L. Chest radiograph ( Fig. 1 ) showed massive right-sided pleural effusion. CECT showed acute pancreatitis with two pancreatic pseudocysts, one at the head of the pancreas (3.8 × 2.8 × 3 cm) and the other anterior to the body (3.7 × 2.4 cm), right-sided massive pleural effusion with a pleuro-pancreatic fistula. Left renal vein thrombosis was also noted ( Fig. 2 ).

**Fig. 3:**
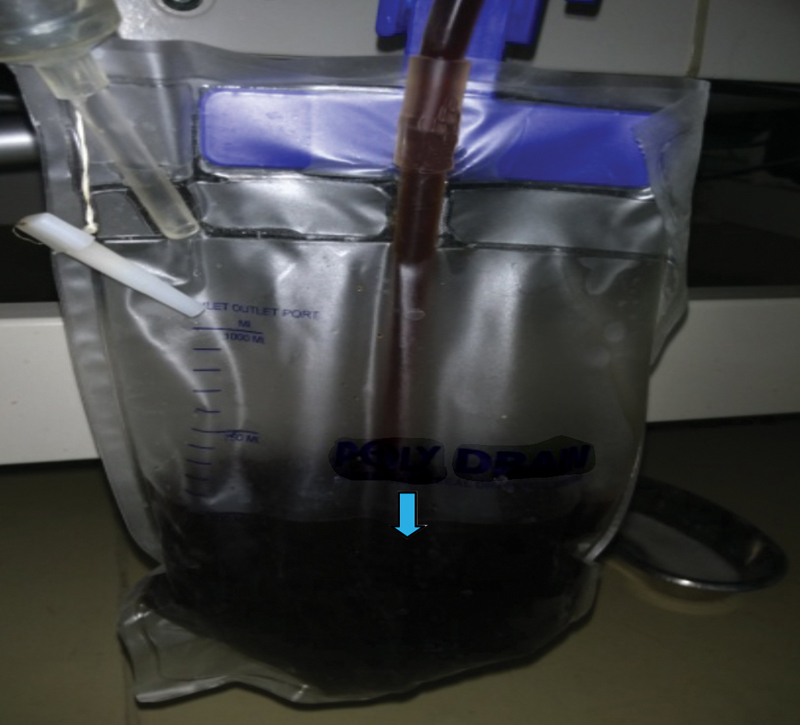
Hemorrhagic pleural fluid.

**Fig. 4:**
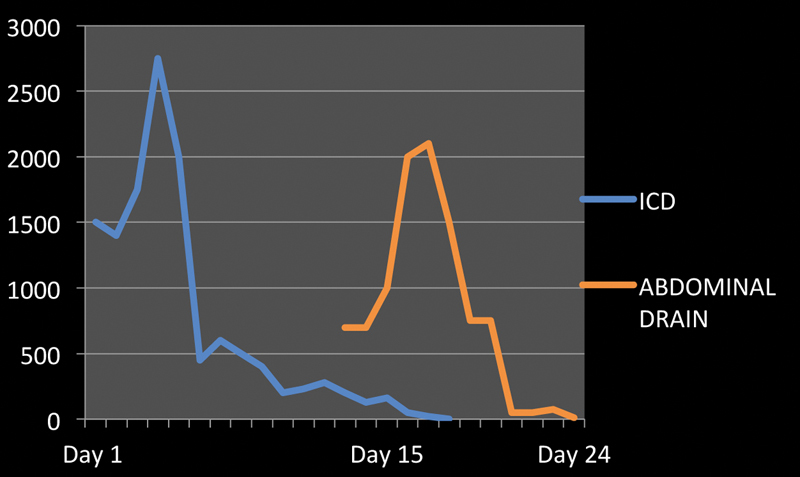
Graph showing chest drain and abdominal drain output (mL).

The patient was admitted to intensive care unit (ICU), resuscitated with fluid, inotropes, and blood transfusions; and was kept on a ventilator after putting in a chest tube, which drained dark brown hemorrhagic pleural fluid ( Fig. 3 ), up to 2 L/d ( Fig. 4 ). His hemoglobin dropped substantially, mandating more blood transfusions. Pleural fluid was exudative, with an elevated amylase level of 10,350 U/L, as against serum levels of 1,253 U/L, confirming the possibility of pleuro-pancreatic communication. He was managed with hemodynamic stabilization, ventilatory support, chest drain, IV antibiotics, and octreotide. The chest drain output initially remained high (up to 2.7 L/d), but subsequently decreased. In 20 days, he was off the ventilator and the drain was also removed.

**Fig. 5:**
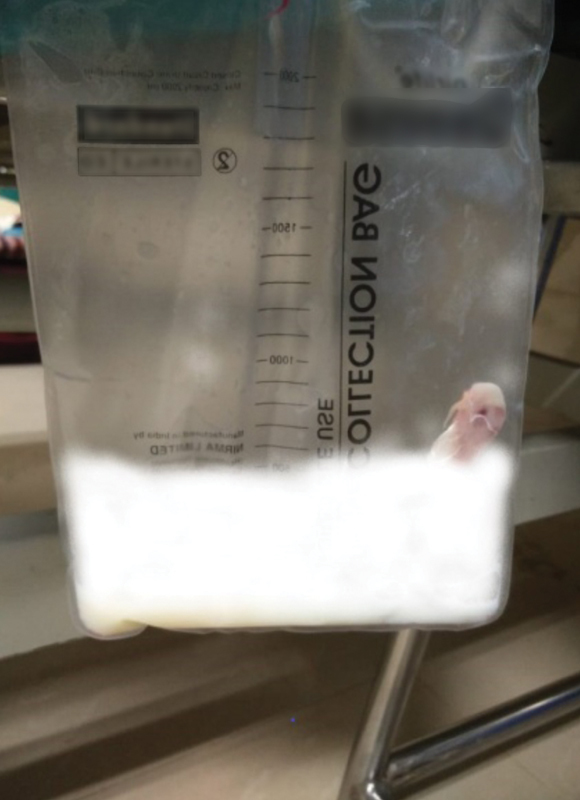
Chylous ascites.

Soon after this, he developed hemorrhagic pancreatic ascites with elevated amylase (713 U/L) and lipase (681 U/L) levels for which an abdominal pigtail drain was placed. Two days later, the pigtail started draining milky white ascitic fluid ( Fig. 5 ) and drain output increased from 700 to 2,100 mL/d ( Fig. 4 ). Biochemical analysis revealed an elevated triglyceride level (703.27 mg/dL) confirming the presence of chylous ascites. He was put on a high protein, low-fat diet, with medium-chain triglycerides (MCTs). Eventually, the drain output also decreased enabling drain removal after 9 days.

He was kept on anticoagulant therapy throughout in view of left renal vein thrombus on CECT. He had a few stormy episodes of sepsis interspersed, but eventually improved and could be discharged after a prolonged stay of 45 days.

## Discussion

Pancreatitis presenting as pleural effusion can be a diagnostic dilemma and needs careful evaluation for the underlying cause. Pathophysiology of pleural effusion in pancreatitis is explained by different mechanisms, one being a mild to moderate reactive effusion with low amylase which resolves spontaneously. Another explanation is the formation of a pancreaticopleural fistula between the pancreatic duct and the pleural cavity, or a large pseudocyst and the pleura, causing massive effusion with high amylase activity. This is difficult to resolve and reaccumulates rapidly after tapping. It occurs in 0.4% of the patients with acute pancreatitis and 4.5% of the patients with associated pseudocysts. 3 Commonly this affects the left side, right-sided effusions accounting for only 19% of all patients. 4 Pleuro-pancreatic fistula has been attributed to duct disruption following repeated pancreatic inflammation or the rupture of a pseudocyst. A posterior duct disruption can track through the esophageal or diaphragmatic hiatus leading to usually a left-sided pleuro-pancreatic fistula, while anteriorly, it connects directly to the peritoneal cavity causing pancreatic ascites. 4 Clinical presentation is often suggestive of the diagnosis. An elevated amylase and lipase level in the pleural fluid is confirmatory when obtained in a setting of acute pancreatitis. Other diagnoses with elevated pleural fluid amylase are esophageal perforation, and certain thoracic malignancies. 5 Apart from chest radiograph, CT scan is useful in providing additional information about the site and size of the effusion, though it may not always be possible to delineate the exact course of the fistula. 6 7 Additional information on the pancreatic pathology and pseudocysts is gained on CT scan. Magnetic resonance cholangiopancreatography (MRCP) is a noninvasive alternative and assists in patients with renal failure, to assess ductal anatomy, disruptions, and fistula course, whereas endoscopic retrograde cholangiopancreatography (ERCP) may prove useful in cases where a stent insertion is contemplated. ERCP and stent placement can relieve the ductal pressure, drain any pancreatic pseudocysts connected to the duct, and can also help in bridging the duct disruption. Operative management is reserved as a second line, though it has also been observed that early operative management can lead to a decreased overall time in fistula closure. 8 The initial line of management is conservative, consisting of thoracocentesis combined with somatostatin analogues to decrease pancreatic secretions.

Pancreatitis accounts for around 4% of all the cases of nontraumatic chylous ascites. 9 Other common causes being lymphatic anomalies, malignancies, liver cirrhosis, and Mycobacterium infection. 9 Pancreatic enzymes are assumed to destroy the lymphatic channels, leading to leakage of chyle into the peritoneum. Another proposed mechanism is blockage of the lymphatics due to inflammation and fibrosis, leading to the exudation of chyle. 10 Diagnosis is made by an increased level of triglycerides in the ascitic fluid, a cut off value of 2 g/L is often advocated by many authors. 11 Management aims to limit chyle flow, to allow healing and closure of the lymphatics. The first line of management is conservative, putting patients on low-fat diets, with MCTs only, and complete avoidance of long-chain triglycerides. As MCTs are directly absorbed and do not contribute to the formation of chylomicrons, they allow the healing of lymphatic-leaks. Somatostatin analogues are often used to decrease the amount of pancreatic secretions. 12 13 Surgical ligation of leaks is reserved for indolent cases.

Twenty-five percent of the patients with pancreatitis have vascular complications, venous complications being less often reported. 14 15 The most common venous complication in pancreatitis is thrombosis of splenic (22%) and portal veins (5.6%). Extra-splanchnic thrombosis is quite rare, though a few authors have reported renal vein thrombosis coexisting with IVC thrombosis. 16 17 Gansbeke and Struyven have reported isolated renal vein thrombosis. 2 The patients are put on anticoagulation and followed up for the resolution of thrombus.

In our patient, there was a combination of three unusual and major life-threatening complications. Simultaneous management of hemodynamic instability, ventilatory demands, sepsis, coagulopathy with massive hemorrhagic effusion, hemorrhagic followed by chylous ascites, as well as anticoagulants for thrombosis was a great challenge. It required a delicate titration of drugs and dosages, meticulous ICU care, multidisciplinary involvement with appropriate nutritional and timely interventional supports, which ultimately rewarded us with a successful outcome. Two years from this incident, the patient is still following up with us. He has put on weight, is off alcohol, and doing well with normal investigation reports.

## Conclusion

The disease course of pancreatitis may be fraught with several complications, some of which might be rare, but contributes to the overall mortality and morbidity. The presence of more than one complication causes additional risk. Prompt diagnosis and judicious management of each complication are warranted for better patient recovery and survival.
